# Development of an Oblique Cone Dielectric Elastomer Actuator Module-Connected Vertebrate Fish Robot

**DOI:** 10.3390/biomimetics10060365

**Published:** 2025-06-04

**Authors:** Taro Hitomi, Ryuki Sato, Aiguo Ming

**Affiliations:** Department of Mechanical and Intelligent Systems Engineering, The University of Electro-Communications, Tokyo 1828585, Japanmingag@uec.ac.jp (A.M.)

**Keywords:** soft robotics, bio-inspired robot, dielectric elastomer actuator, oblique cone DEA, modeling

## Abstract

As a soft actuator for fish robots, an oblique cone dielectric elastomer actuator (DEA) module inspired by the structure of white muscles in fish was proposed in the authors’ previous study. However, a mathematical model of an oblique cone DEA was not established, and designing a drive module that took into account its driving characteristics and passivity for integration into a fish robot remained a challenge. The purpose of this paper is to develop a vertebrate fish robot using multiple oblique cone DEA modules to achieve fish-like bending capability. First, an oblique cone DEA module was modeled for the design of a fish robot. The relationships among bending angle, blocking torque, driving voltage, and design parameters were established and confirmed by comparing the calculated and experimental results. Based on the modeling results, we designed an oblique cone DEA module-connected vertebrate fish robot. Finally, the experimental results of the fabricated fish robot demonstrated that the model-based design enabled flexible body swinging and swimming through a multiple-module-connected vertebrate structure.

## 1. Introduction

The demand for underwater robots is increasing in fields such as marine research, underwater equipment maintenance, and inspection. Most underwater robots currently in practical use are driven by screw propellers. However, these are difficult to control when swimming along complex routes. Propeller-type robots also have problems with cavitation and noise, and can become entangled with plants and other objects, posing a danger to sea life. On the other hand, fish are able to swim skillfully in a flowing environment without damaging their surroundings. For this reason, biomimetic fish robots that focus on the way fish swim are attracting attention. Research and development on fish robots that imitate the swimming behavior of fish have been actively conducted [1,2,3]. However, most of them relied on rotary motors with a hard shell made of metallic material.

Soft materials are particularly well-suited for replicating the undulating body movements of fish, and studies on soft fish robots using soft actuators have increased in number in recent years. Some of the fish robots that have been developed include those that use actuators that deform due to air pressure or fluid pressure [4], those that use Shape Memory Alloys (SMAs) [5], those that use hydraulically amplified self-healing electrostatic (HASEL) actuators [6], those that use Macro Fiber Composites (MFCs) [7,8,9], and those that use dielectric elastomer actuators (DEAs) [10,11]. However, these robots exhibit a small amount of deformation and are unable to replicate the swimming shape of fish, resulting in a slow swimming speed. This is due to the small amount of deformation of the soft actuator and the small generated force. Furthermore, although some existing soft fish robots, including those referenced above, utilize multiple soft actuators in parallel, they, along with many others, typically only have one or two actuators in series along their body axis.

To address the performance limitations of soft fish robots due to the low output of soft actuators, we focused on the anatomical structure and muscle properties of fish. By developing a drive module that mimics the structure of a muscle, we aimed to enhance the deformation capacity of the soft actuator, thereby realizing a fish-like swimming shape. In our previous study, we developed an oblique cone DEA drive module that mimics the white muscle of a fish [12]. It has been experimentally demonstrated that the bending capacity can be enhanced by shifting the apex of the cone DEA. However, as the relationship between the drive module’s design parameters and the output was not clearly defined, understanding the drive performance required actual fabrication. Consequently, the design of a drive module specifically for a fish robot has not been undertaken. In this paper, we present the modeling of an oblique cone DEA and a drive module, enabling the examination of the impact of the design parameters on the bending capacity of the module. By designing a drive module based on the modeling, we developed a prototype of a soft fish robot using multiple oblique cone DEA modules. The main contribution of this paper is the modeling and identification of an oblique cone DEA module taking into account hyperelasticity, and the integration of multiple drive modules designed using modeling into a biomimetic soft fish robot by connecting them in series along the robot’s body axis.

The remains of this paper are organized as follows. Section 2 describes the structure of the oblique cone DEA module that mimics the white muscle of fish. Section 3 presents the modeling of the drive module. Section 4 describes the design of a fish robot that connects multiple oblique cone DEA modules. Section 5 presents the fabrication of the fish robot and the results of its performance verification experiments. Section 6 concludes the paper.

## 2. Structure of White Muscle in Fish and Oblique Cone DEA Module

### 2.1. Structure of White Muscle in Fish

The muscles constituting a fish’s body are broadly divided into white muscle and red muscle, with white muscle being primarily recruited for large body flexions during rapid turns and sudden starts. Based on this functional aspect of fish biology, we considered that by mimicking the white muscle structure in a fish robot, the force and displacement of soft actuators could be effectively utilized for the robot’s flexion.

The white muscles of fish, which have an oblique cone shape, are located bilaterally along the spine [13]. These muscles often work antagonistically during bending motions. The apex of the oblique cone is situated closer to the spine, a structure that allows for an efficient transmission of flexion force to the spine. If the white muscle on either the left or right side stretches and the antagonistic action is lost, the body flexes. Based on these characteristics, the development of a module using an oblique cone DEA to perform bending motion was undertaken.

### 2.2. Oblique Cone DEA and Module

In this study, we employed DEA to fabricate a module that mimics the oblique cone shape of white muscle. DEA was selected for two reasons: first, DEA has a high electromechanical conversion rate and a large strain rate, which allows for large bending; second, it is flexible and can be easily fabricated into a variety of shapes. In fact, many different shapes of DEA have been developed until now, including conical DEAs [14,15,16,17]. The modeling of planar DEAs and cone DEAs has also been conducted, and the validity of their model has been demonstrated in previous studies, including [17]. However, it should be noted that, to our knowledge, no study exists on oblique cone DEAs characterized by a planar offset apex, other than our work, and that no mathematical model has yet been established. Thus, to apply this concept to a fish robot, it is necessary to both model the drive modules and design the modules considering their integration into the vertebrate fish robot.

**Figure 1:**
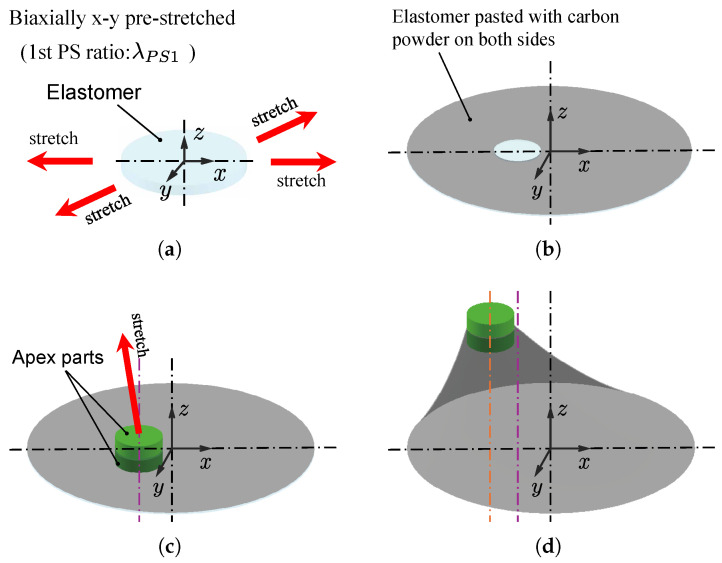
Fabrication procedure of the oblique cone DEA. (**a**) State before 1st PS; (**b**) state after 1st PS; (**c**) state before 2nd PS; (**d**) state after 2nd PS.

Figure 1 shows the fabrication procedure of an oblique cone DEA. First, the elastomer, VHB Y-4905J (3M, USA), was biaxially pre-stretched in the planar direction and attached to the edge of a hole of a certain radius in the lower base frame part. Then, carbon powder as a flexible electrode was pasted to both surfaces of the elastomer, except where the apex parts were to be connected. Next, the apex part of the oblique cone was connected to the elastomer. Finally, the oblique cone shape was fabricated by lifting the apex part obliquely upward. In the fabrication process of the oblique cone DEA, the elastomer was pre-stretched twice: initially in a planar direction, and subsequently as the apex was lifted upward at an oblique angle. In this paper, we refer to them as the first-stage pre-stretch (1st PS) and the second-stage pre-stretch (2nd PS), respectively. Introducing two offsets during the fabrication of states (c) and (d) in Figure 1, along with diagonally lifting the apex upwards in the 2nd PS to ensure a consistent resilient force across the generatrix, helps reduce the necking of the generatrix in the oblique cone DEA. This prevents non-uniform necking on the circumference, thereby minimizing modeling error and the deterioration of the driving performance.

**Figure 2:**
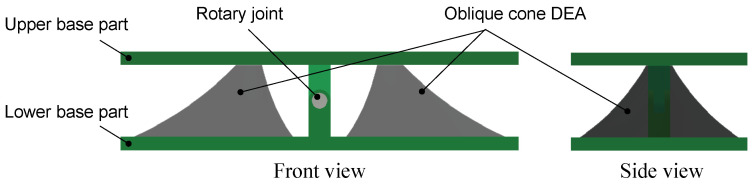
The structure of the oblique cone DEA module.

Next, we explain the structure of the oblique cone DEA drive module illustrated in Figure 2. The module consists of two oblique cone DEAs that mimic white muscle and are in an antagonistic state. The upper and lower parts mimic fascia, and the axis of rotation mimics a joint. The apex part of the oblique cone DEA attached to the lower base part is lifted to form the oblique cone and fixed to the upper base part. This allows the DEA to maintain its form of oblique cone shape. The upper base part holds the lower base frame part of the next module. In other words, the DEA of the next module is attached to the upper base part of the module. By stacking and connecting these modules together, a vertebrate structure robot can be formed.

**Figure 3:**
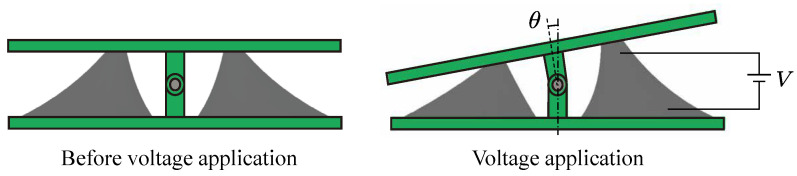
The driving principle of the oblique cone DEA module.

Figure 3 shows the driving principle of the drive module. Initially, the left and right oblique cone DEAs are antagonistic to each other across the axis of rotation. When voltage is applied to one side of the DEA, the resilience of the DEA on the applied side decreases due to Maxwell stress. This causes the left and right antagonism to collapse, and the module bends until the left and right DEAs’ antagonism is balanced again. When the application of voltage is stopped, the module returns to its initial state.

## 3. Modeling of Oblique Cone DEA Modules for Design

### 3.1. Model and Design Parameters

**Figure 4:**
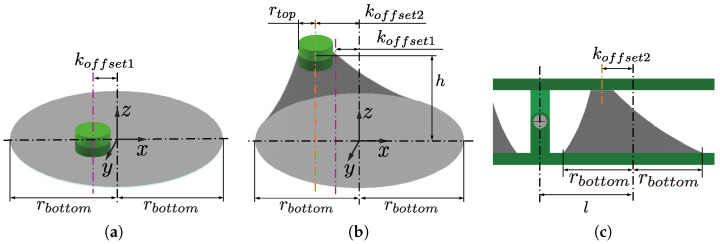
The design parameter of the oblique cone DEA module. (**a**) State before 2nd PS; (**b**) state after 2nd PS; (**c**) front view of the module.

Figure 4 shows the definition of the design parameters of the drive module. The module has eight parameters: the radius of the top and bottom circles of the oblique cone, $r_{top}$ [mm] and $r_{bottom}$ [mm], respectively; the distance between the center axes of the apex part and the bottom circle in the state before and after 2nd PS, $k_{offset1}$ [mm] and $k_{offset2}$ [mm], respectively; the horizontal distance between the center axis of the oblique cone module and the center axis of the bottom circle *l* [mm]; the height of the oblique cone DEA, *h* [mm]; the initial thickness of the elastomer, $d_{0}$ [mm]; and the 1st PS ratio, $\lambda_{PS1}$.

### 3.2. Calculation of Output Bending Angle and Blocking Torque

**Figure 5:**
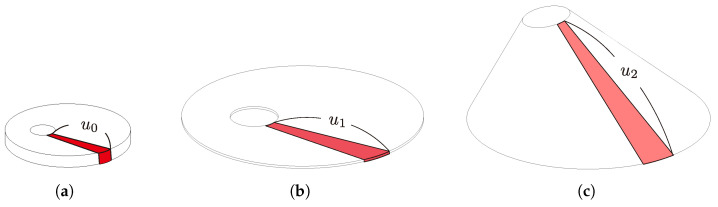
The deformation process of the infinitesimal areas. (**a**) state before 1st PS; (**b**) state before 2nd PS; (**c**) state when the module is bent.

As shown in Figure 5, the oblique cone DEA is divided into infinitesimal areas, and we focus on its deformation process. $u_{0}$, $u_{1}$, and $u_{2}$ are defined for the lengths of the generatrix of the infinitesimal areas before the 1st PS, before the 2nd PS, and in the module bending state, respectively.

**Figure 6:**
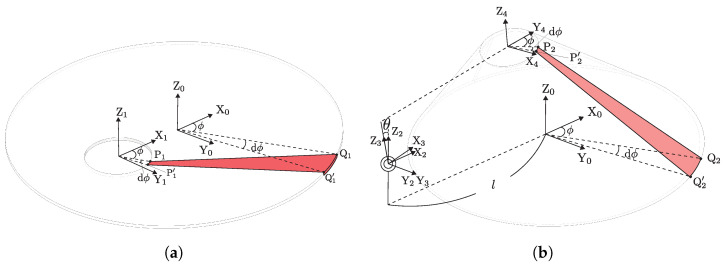
Coordinates of each point in the infinitesimal area. (**a**) State before 2nd PS; (**b**) state when the module is bent.

Figure 6 show the infinitesimal areas at the state before 2nd PS and when the module is bent by θ. The infinitesimal area is a domain enclosed by the circumferences of the upper and lower circles, divided by a slight angle dϕ relative to their respective central axes.

The coordinates of $P_{1}$ and $Q_{1}$ in coordinate system $\Sigma_{0}$ are given by

(1) $$P_{1}=\left[\begin{array}{c} r_{top}\cos\phi \\ r_{top}\sin\phi \\ 0 \end{array}\right]+\left[\begin{array}{c} -k_{offset1}\\ 0\\ 0 \end{array}\right],$$

(2) $$\begin{array}{cc} Q_{1} & =\left[\begin{array}{c} r_{bottom}\cos\phi \\ r_{bottom}\sin\phi \\ 0 \end{array}\right]. \end{array}$$

Therefore, $u_{1}$ is given by

(3) $$\begin{array}{c} u_{1}\left(\phi \right)=\left|\vec{P_{1}Q_{1}}\right|. \end{array}$$

Then, $u_{0}$ can be calculated as

(4) $$\begin{array}{c} u_{0}\left(\phi \right)=\frac{u_{1}\left(\phi \right)}{\lambda_{PS1}}. \end{array}$$

The coordinates of point $P_{2}$ is given by

(5) $$P_{2}=\left[\begin{array}{c} -l\\ 0\\ \frac{h}{2} \end{array}\right]+\left[\begin{array}{ccc} \cos\theta & 0 & \sin\theta \\ 0 & 1 & 0\\ -\sin\theta & 0 & \cos\theta \end{array}\right]\left[\left[\begin{array}{c} l-k_{offset2}\\ 0\\ \frac{h}{2} \end{array}\right]+\left[\begin{array}{c} r_{bottom}\cos\phi \\ r_{bottom}\sin\phi \\ 0 \end{array}\right]\right].$$

Therefore, $u_{2}$ is given by

(6) $$\begin{array}{c} u_{2}\left(\phi ,\theta \right)=\left|\vec{P_{2}Q_{2}}\right| \end{array}$$

Note that Point $Q_{2}$ is fixed in position after 1st PS, so its coordinates are equal to $Q_{1}$.

**Figure 7:**
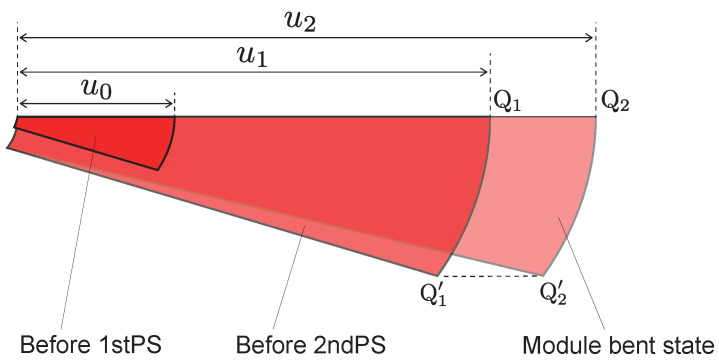
Comparison of infinitesimal areas before 1st PS and 2nd PS and in the module bending state.

A comparison of infinitesimal areas before 1st PS and 2nd PS and in the module bending state is shown in Figure 7. The stretching ratio in the direction of the generatrix, $\lambda_{r}$, is given by

(7) $$\begin{array}{c} \lambda_{r}\left(\phi ,\theta \right)=\frac{u_{2}\left(\phi ,\theta \right)}{u_{0}}. \end{array}$$

There is no extension in the circumferential direction after the 2nd PS. Therefore, the circumferential stretch ratio $\lambda_{c}$ can be expressed by

(8) $$\begin{array}{c} \lambda_{c}=\lambda_{PS1}. \end{array}$$

The material is hypothesized to be incompressible, meaning its volume remains constant before and after deformation. Thus, the stretching ratio in the thickness direction, $\lambda_{t}$, is given by

(9) $$\begin{array}{c} \lambda_{t}\left(\phi ,\theta \right)=\frac{1}{\lambda_{r}\left(\phi ,\theta \right)\lambda_{c}}. \end{array}$$

The stress in the generatrix direction in an infinitesimal area in the module bending state, $\sigma_{r}$, can be calculated using the Yeoh model as

(10) $$\begin{array}{c} \sigma_{r}\left(\phi ,\theta \right)=2\left(\lambda_{r}^{2}\left(\phi ,\theta \right)-\frac{2}{\lambda_{r}\left(\phi ,\theta \right)}\right)\sum_{i=1}^{3}iC_{i}\left(\lambda_{r}^{2}\left(\phi ,\theta \right)+\frac{2}{\lambda_{r}\left(\phi ,\theta \right)}-3\right), \end{array}$$

where $C_{1}$, $C_{2}$, and $C_{3}$ are the material constants of the hyperelastic model.

Maxwell stress acts on the voltage-applied DEA [17]. The stresses in the generatrix direction on the voltage-applied side DEA, $\sigma_{r,on}$, and the voltage-unapplied side DEA, $\sigma_{r,off}$, are calculated as

(11) $$\begin{array}{cc} \sigma_{r,on}\left(\phi ,\theta \right) & =\sigma_{r}\left(\phi ,\theta \right)-\frac{1}{2}\varepsilon_{0}\varepsilon_{r}{\left(\frac{V}{\lambda_{t}d_{0}}\right)}^{2}, \end{array}$$

(12) $$\begin{array}{cc} \sigma_{r,off}\left(\phi ,\theta \right) & =\sigma_{r}\left(\phi ,\theta \right). \end{array}$$

Therefore, the force that the infinitesimal area exerts on the apex part is expressed as

(13) $$\begin{array}{cc} dF_{on}\left(\phi ,\theta \right) & =\sigma_{r,on}\left(\phi ,\theta \right)r_{top}d\phi \lambda_{t}\left(\phi ,\theta \right)d_{0}, \end{array}$$

(14) $$\begin{array}{cc} dF_{off}\left(\phi ,\theta \right) & =\sigma_{r,off}\left(\phi ,\theta \right)r_{top}d\phi \lambda_{t}\left(\phi ,\theta \right)d_{0}, \end{array}$$

where $r_{top}d\phi$ represents the length of the top circle when its circumference is divided by a small angle dϕ and $\lambda_{t}\left(\phi ,\theta \right)d_{0}$ represents the thickness of the elastomer when the module is bent by θ. Taking into account the direction of the forces, the respective force vectors are obtained as

(15) $$\begin{array}{cc} dF_{on}\left(\phi ,\theta \right) & =\frac{\vec{P_{2}Q_{2}}}{|\vec{P_{2}Q_{2}}|}dF_{on}\left(\phi ,\theta \right) \end{array}$$

(16) $$\begin{array}{cc} dF_{off}\left(\phi ,\theta \right) & =\frac{\vec{P_{2}Q_{2}}}{|\vec{P_{2}Q_{2}}|}dF_{off}\left(\phi ,\theta \right) \end{array}$$

The moment arm corresponding to the infinitesimal area corresponds to the coordinate of point $P_{2}$ in coordinate system $\Sigma_{2}$ as shown in Figure 6b, and the moment arms on the voltage-applied side $L_{on}$ and the voltage-unapplied side $L_{off}$ expressed as

(17) $$L_{on}\left(\phi ,\theta \right)=\left[\begin{array}{ccc} \cos\theta & 0 & \sin\theta \\ 0 & 1 & 0\\ -\sin\theta & 0 & \cos\theta \end{array}\right]\left[\left[\begin{array}{c} l-k_{offset2}\\ 0\\ \frac{h}{2} \end{array}\right]+\left[\begin{array}{c} r_{bottom}\cos\phi \\ r_{bottom}\sin\phi \\ 0 \end{array}\right]\right],$$

(18) $$L_{off}\left(\phi ,\theta \right)=\left[\begin{array}{ccc} \cos\left(-\theta \right) & 0 & \sin\left(-\theta \right)\\ 0 & 1 & 0\\ -\sin\left(-\theta \right) & 0 & \cos\left(-\theta \right) \end{array}\right]\left[\left[\begin{array}{c} l-k_{offset2}\\ 0\\ \frac{h}{2} \end{array}\right]+\left[\begin{array}{c} r_{bottom}\cos\phi \\ r_{bottom}\sin\phi \\ 0 \end{array}\right]\right].$$

Thus, the moments of the infinitesimal area of the voltage-applied $dM_{on}$ and voltage-unapplied $dM_{off}$ sides are given by

(19) $$\begin{array}{cc} dM_{on}\left(\phi ,\theta \right) & =dF_{off}\left(\phi ,\theta \right)\times L_{on}\left(\phi ,\theta \right), \end{array}$$

(20) $$\begin{array}{cc} dM_{off}\left(\phi ,\theta \right) & =dF_{off}\left(\phi ,\theta \right)\times L_{off}\left(\phi ,\theta \right). \end{array}$$

By integrating the infinitesimal areas over the entire circumference, the moment of the entire oblique cone DEA can be obtained as

(21) $$\begin{array}{cc} M_{on}\left(\theta \right) & =\int_{0}^{2\pi }dM_{on}\left(\phi ,\theta \right), \end{array}$$

(22) $$\begin{array}{cc} M_{off}\left(\theta \right) & =\int_{0}^{2\pi }dM_{off}\left(\phi ,\theta \right). \end{array}$$

The bending angle of the module can be calculated by finding θ that satisfies the following equation.

(23) $$\begin{array}{c} M_{on}\left(\theta \right)+M_{off}\left(\theta \right)=0. \end{array}$$

The blocking torque at a bending angle of 0 degrees, $\tau_{0}$, can be obtained by

(24) $$\begin{array}{c} \tau_{0}=M_{on}\left(0\right)+M_{off}\left(0\right). \end{array}$$

### 3.3. Identification of Material Parameters

**Figure 8:**
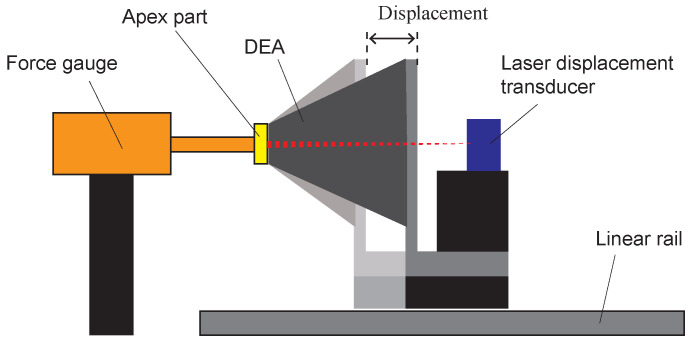
Tensile test method.

To identify the material constants of DEA, we conducted the tensile tests using a fabricated cone DEA. Equation (10) is used to determine the hyperelastic material parameters from the measured forces and displacements. Figure 8 illustrates the tensile test method. A force gauge (FGP-0.5, Nidec, Kyoto, Japan) was used to measure force, and a laser displacement transducer (IL-S100, KEYENCE, Osaka, Japan) was used to measure displacement. The cone DEA was placed horizontally on a motorized linear stage (SGAMH26-200, SIGMA KOKI, Saitama, Japan) with its central axis coinciding with the measurement axis of the force gauge. The linear stage was controlled at a constant speed of 0.05 mm/s with a cone height ranging from 2 mm to 37 mm. In this experiment, the DEA was not applied to voltage. Measured data acquisition from the force gauge and laser sensor, as well as control of the linear stage, were all implemented in LabVIEW.

The resilient force of the DEA acting on the force gauge can be calculated from Equation (16) as follows:

(25) $$\begin{array}{c} F=\int_{0}^{2\pi }dF_{off}\left(\phi ,\theta \right) \end{array}$$

**Figure 9:**
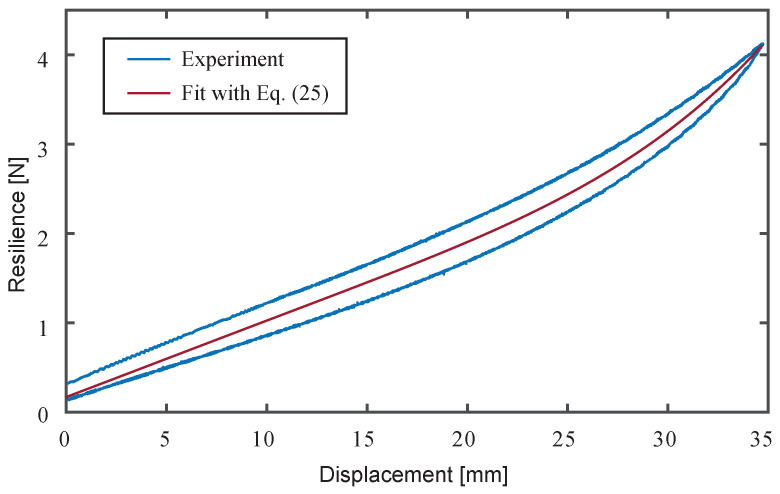
Result of the tensile test.

The results of the tensile test of the cone DEA and the fitting results using the least squares method based on Equation (25) are shown in Figure 9. The horizontal axis of the graph represents the displacement of the cone DEA height, as defined in Figure 8, and displacement = 0 mm corresponds to a cone DEA height of 2 mm. The material constants of the hyperelastic model identified from the fitting results are listed in Table 1.

### 3.4. Output Comparison Between Calculated Results and Experimental Results

To verify the validity of the modeling, we fabricated a prototype drive module and conducted driving experiments. The design parameter combination of the prototyped drive modules for the output comparison experiments is listed in Table 2. In this experiment, we varied the combinations of $k_{offset1}$ and $k_{offset2}$ to form the oblique cone, the feature of this study, while fixing all other parameters. The combinations of $k_{offset1}$ and $k_{offset2}$ were adjusted so that $\lambda_{PS2}$ was constant for 0≤ϕ≤2π.

**Figure 10:**
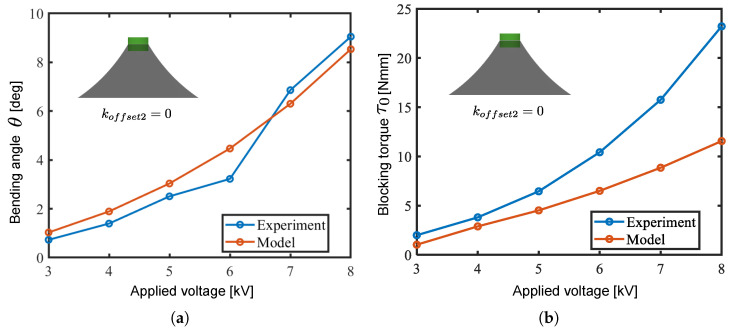
Output comparison between the model and experiment using a drive module with pure cone DEAs. (**a**) Bending angle; (**b**) blocking torque.

**Figure 11:**
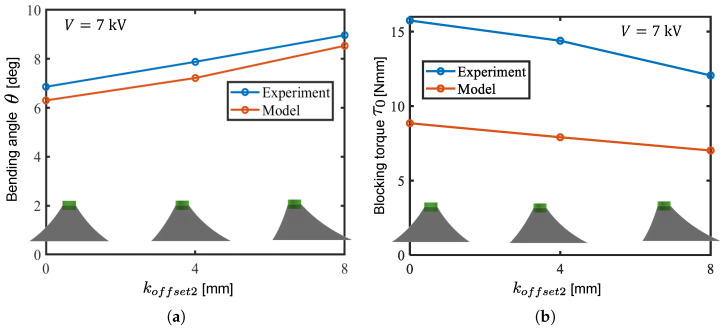
Output comparison between the models and experiments with varied $k_{offset2}$. (**a**) Bending angle; (**b**) blocking torque.

Figure 10 shows the relationship between the applied voltage and the bending angle and blocking torque obtained in calculation and experiment using a drive module with pure cone DEAs with $\left(k_{offset1},k_{offset2}\right)=\left(0,0\right)$. Figure 11 shows the relationship between the $k_{offset2}$ and the bending angle as well as blocking torque obtained in the calculation and experiment. Comparing the calculated and experimental results, the fluctuations in the module angle and blocking torque with respect to the applied voltage and $k_{offset2}$ were consistent. The results in Figure 11 indicate that forming oblique cone DEAs with $k_{offset2}$ improves the bending angle output of the module.

One of the reasons for the discrepancy in absolute values between the simulations and experiments can be considered as being that the necking of the cone DEA’s generatrix was not considered in the calculations. In the model, the generatrix of each infinitesimal segment was assumed to be straight; however, in the actual DEA, necking occurs after the 2nd PS due to the difference in stretch ratios between the generatrix and circumferential directions. Accordingly, this study focused on the trends in output characteristics caused by variations in model parameters and applied these trends to the design of the robot.

## 4. Design of a Fish Robot Using Multiple Oblique Cone DEA Modules

### 4.1. Basic Policy for Design

The mechanical properties of fish muscle are known to vary depending on the anatomical region [18]. We are focusing on the observation that muscle strain increases from the head towards the tail. From this observation, we can infer that angular velocity likely also increases from the head to the tail. On the other hand, it has also been observed that muscle power output decreases from the head to the tail. Combining these observations leads to the assumption that vertebral torque is greater closer to the head. Consequently, we adopted a basic design policy for the drive modules of the fish robot: to design modules such that the torque generated is greater towards the head.

**Figure 12:**
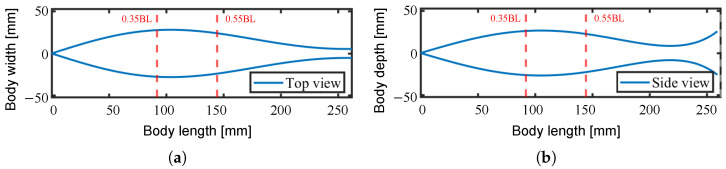
The abstract form of the fish robot. (**a**) Body width; (**b**) body depth.

The abstract form, specifically the body width and depth, of the robot was determined and scaled based on the mathematical model of a tuna designed by Alvarado [19] as shown in Figure 12. In other words, the width of each module is subject to different constraints based on its location. This approach, unlike our prior more uniformly shaped fish robot developed in [12], allows for a more biomimetic form.

We determined the total length of the fish robot *L* to 262 mm, taking into account the feasible size for manufacturing the drive modules. Accordingly, we decided to place multiple drive modules in the range of 0.35 to 0.55 body length (BL), as this area is typically highly active during propulsion.

### 4.2. Details of Design

Our design procedure for the multiple drive modules involved calculating all possible combinations of the design parameters of the module using the developed model equations. Based on these calculation results, we then determined which module to use for each position.

The ranges for the design parameters are summarized in Table 3. However, combinations that violated the manufacturing constraints shown in Equations (26)–(28) were excluded. These constraints are put in place to secure the carbon powder coating area, secure the distance between the slanted cone DEA and the links, and maintain the slanted cone shape.

(26) $$\begin{array}{ccc} r_{bottom}-\left(r_{top}+k_{offset1}\right) & > & 4\;\left[\mathrm{mm}\right] \end{array}$$

(27) $$\begin{array}{ccc} l & \geq & r_{bottom}+5\;\left[\mathrm{mm}\right] \end{array}$$

(28) $$\begin{array}{ccc} r_{bottom} & \geq & r_{top}+k_{offset2} \end{array}$$

**Figure 13:**
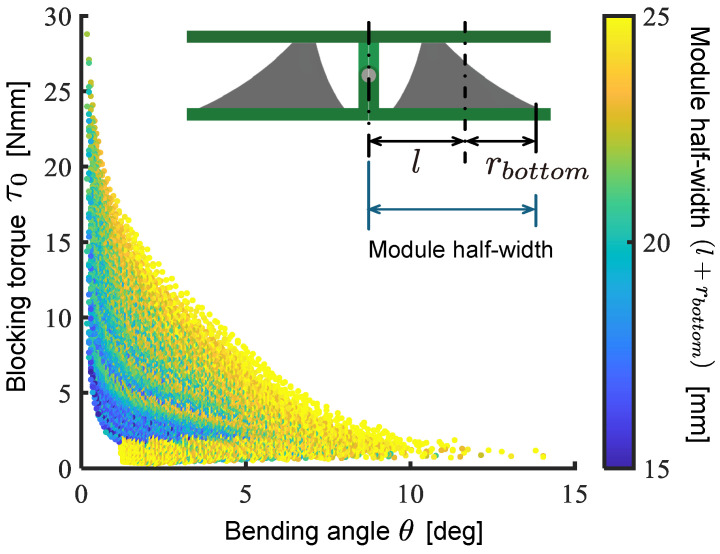
Calculation result of design parameter sweep.

Figure 13 shows a plot illustrating the relationship between the maximum bending angle and blocking torque of the modules based on the results of calculating all possible combinations. The results indicate a trade-off relationship between the bending angle and blocking torque of the drive module, which aligns with the module characteristics suggested in our previous work. Furthermore, it is clear that a smaller module width leads to a reduction in both the bending angle and the blocking torque.

Consequently, we determined the arrangement of six modules within the range of 0.35 BL to 0.55 BL as shown in Table 4, aiming for a balanced bending angle and blocking torque, with the torque increasing from the tail towards the head. The modules were assigned numbers ➀ to ➅ from the tail side to the head side.

## 5. Fabrication and Experimental Results

### 5.1. Fabrication

**Figure 14:**
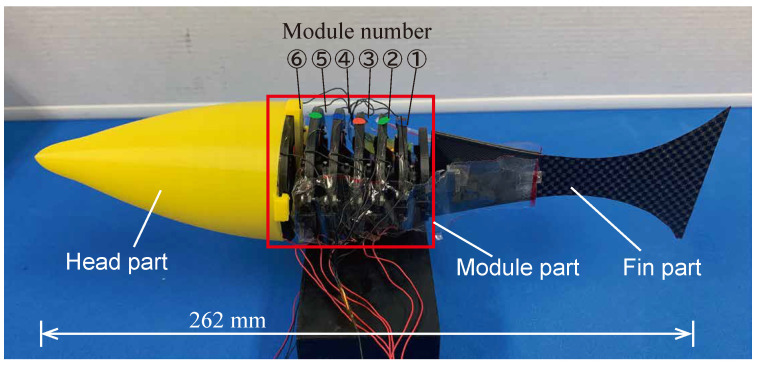
Prototype of fish robot.

Figure 14 shows the fish robot fabricated based on the design policy. The fish robot was fabricated by connecting the designed six-drive modules and connecting the head and tail fin parts at the front and rear. The head part and the upper and lower base frame parts of the drive modules were made of Poly-Lactic Acid (PLA) resin using 3D printing. The fin part was made from a thin Carbon Fiber-Reinforced Plastic (CFRP) plate and plastic pieces. A vinyl sheet was applied from both sides of the modules to the tail fin to reduce the fluid resistance of the modules.

**Figure 15:**
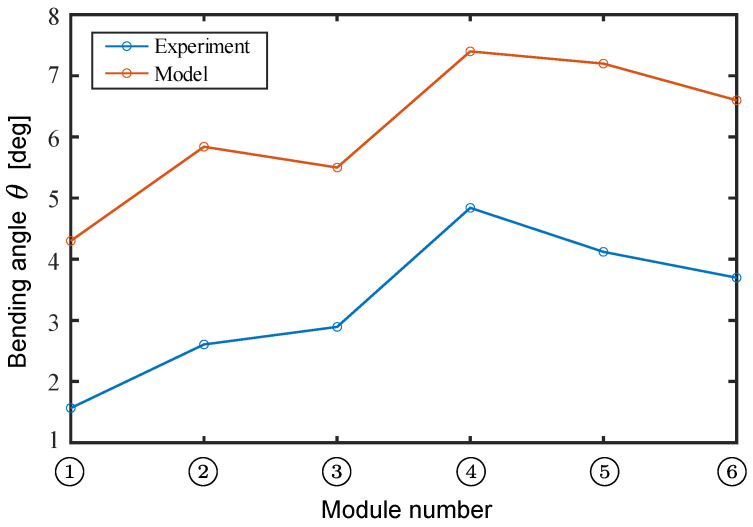
Experimental and calculated bending angles for each module.

First, we conducted experiments to verify the performance of each fabricated module, measuring the bending angle. The left and right oblique cone DEAs were actuated using a 0.2 Hz low-frequency sinusoidal voltage, ranging from 0 kV to 6 kV, with a phase difference of π rad. The experimental and model calculation results are shown in Figure 15.

The maximum bending angle of 4.84 deg was recorded by Module ➃ in the experiment. The correlation coefficient with the model calculation results using Equation (29) is r=0.97, indicating that the calculated trend of the bending angle by the model reproduced the trend of the actual module with high accuracy.

(29) $$r=\frac{\sum \left(x_{i}-\bar{x}\right)\left(y_{i}-\bar{y}\right)}{\sqrt{\sum {\left(x_{i}-\bar{x}\right)}^{2}\sum {\left(y_{i}-\bar{y}\right)}^{2}}},$$

where i=1,2,...,6 is the data index, $x_{i}$ and $y_{i}$ represent the experimental and model calculation data, respectively, and $\bar{x}$ and $\bar{y}$ represent the means of experimental and model calculation data, respectively.

**Figure 16:**
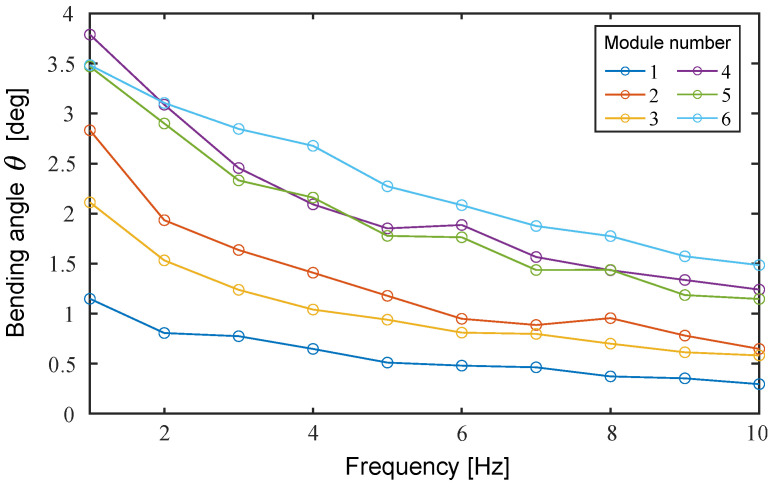
Frequency response characteristics of bending angle.

Next, we verified the frequency response characteristics of the bending angle. The left and right oblique cone DEAs were actuated using a 1 Hz to 10 Hz sinusoidal voltage, ranging from 0 kV to 6 kV, with a phase difference of π rad. As shown in Figure 16, the bending angles of Modules ➃ and ➅ are reversed at a frequency of lower than 2 Hz. The smaller rate of decrease in the bending angle of Module ➅ is inferred due to the relatively large blocking torque. Therefore, the amount of blocking torque is considered crucial in the high-frequency band.

### 5.2. Experiments in Air

**Figure 17:**
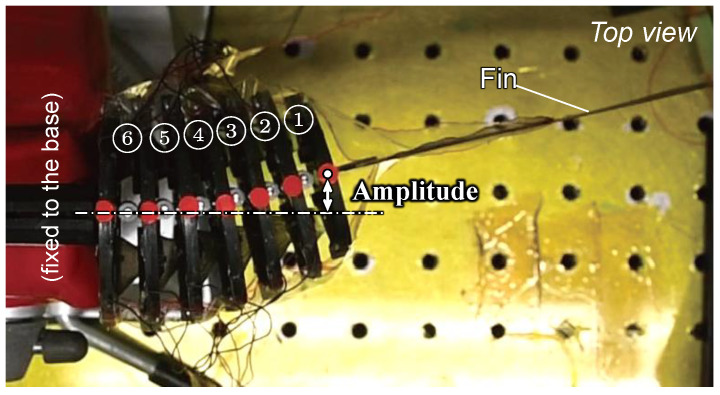
Experimental setup for fin swing in air.

To evaluate the performance of the fish robot, we first conducted an experiment where the connected modules were swung in the air. The six modules were connected and fixed together, and the front half body was fixed to the base as shown in Figure 17. The right and left oblique cone DEAs of every module were actuated by a 0 kV to 6 kV amplitude and a 1 Hz to 10 Hz sinusoidal voltage with a π rad phase difference.

**Figure 18:**
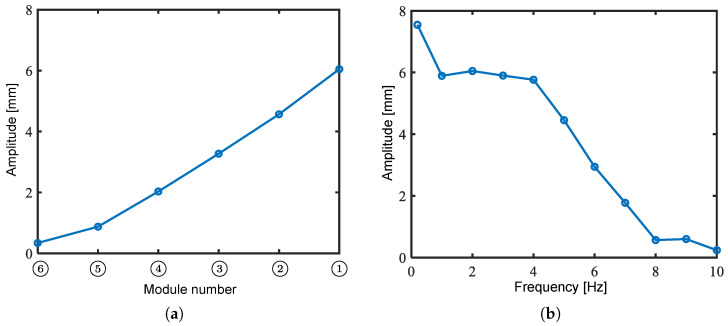
Experimental results of fin swing in air. (**a**) Amplitude of each module at driving frequency of 2 Hz; (**b**) relationship between driving frequency and amplitude of Module ➀.

Figure 18 shows the experimental results of the relationship between the frequency of the applied voltage and the amplitude of the swing of Module ➀. Figure 18a clearly shows that the amplitude increase rate increases monotonically from Module ➅ to ➀, promising the formation of a smooth swimming shape that is less susceptible to fluid resistance. As shown in Figure 18b, the amplitude decreased gradually between 2 Hz and 4 Hz. The maximum amplitude of 6.05 mm was recorded at 2 Hz, excluding the very slow drive frequency of 0.2 Hz. These results indicate a resonant frequency near 2 Hz, attributed to module connections. The limited frequency response of the modules likely causes the sharp amplitude decline above 4 Hz.

### 5.3. Experiments in Insulating Fluid

The prototyped fish robot was made to swim in insulating fluid Fluorinert (FC-283, 3M, USA) to investigate swimming speed and swimming shape. A 0 kV to 10 kV amplitude sinusoidal voltage was applied to the DEAs. The swimming speed and shape of the robot were obtained by tracking images captured by a camera fixed on the top of the tank.

**Figure 19:**
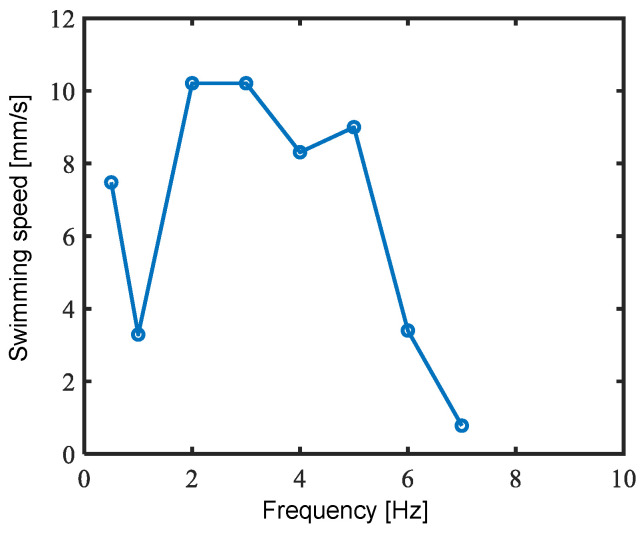
Experimental results of swimming speed at varying driving frequencies.

Figure 19 illustrates the variation in swimming speed with the frequency of the applied voltage waveform. The maximum speed of 10.2 mm/s, where its body length ratio speed was 0.04 BL/s, was recorded at the driving frequency of 2 and 3 Hz. In experiments where the driving frequency was 7 Hz or higher, the amplitude of the tail fin decreased significantly, and propulsion was not possible.

**Figure 20:**
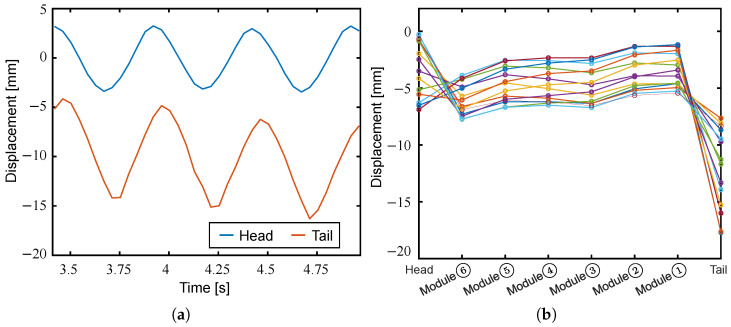
Experimental results of swimming in fluid at a driving frequency of 2 Hz. (**a**) Head and tail displacements; (**b**) swimming shape.

Figure 20 shows the head and tail displacements for three periods and the swimming shape for one period at a driving frequency of 2 Hz. As shown in Figure 20a, the amplitude of the tail fin was larger than that of the head, and it is confirmed that the swimming shape was achieved in accordance with the elongate body theory, as shown in Figure 20b. However, the increase in amplitude from the head to the tail in the span where modules were arranged was relatively small, and the amplitude decreased at Module ➁. This may be because the torque generated by the modules was small and the bending angles decreased due to the fluid resistance. To improve the swimming speed of the robot, it is desirable to select modules designed with larger blocking torques. Stacking oblique cone DEAs would be one reasonable solution for increasing power density in a limited space.

## 6. Conclusions

In this paper, we developed an oblique cone DEA module-connected vertebrate fish robot. To apply this interconnected drive module concept to a fish robot, we established a mathematical model for module design. This model clearly describes the relationship between the design parameters and the output performance. Based on this model, we developed a prototype of a vertebrate fish robot with multiple specifically designed drive modules. In experiments conducted in air, we validated the modeling and model-based design, confirming that the bending ability increases along the body length, similar to that of a fish. In the experiments in fluid, we confirmed that the developed vertebrate fish robot is capable of swimming by wiggling its body. Future work includes an analysis of motion in fluids and an improvement of the performance of the drive module. A dynamic model of the module is necessary for analyzing motion in fluids; therefore, improvements to the model are required. In addition, the drive module will require an increase in DEA density and the number of connections.

## Figures and Tables

**Table 1:** Material constants identified by tensile test.

$C_{1}$ [MPa]	$C_{2}$ [MPa]	$C_{3}$ [MPa]
$3.65\times 10^{-2}$	$-1.11\times 10^{-4}$	$1.98\times 10^{-6}$

**Table 2:** Design parameter combination of the prototyped drive modules for the output comparison experiments.

($k_{\mathrm{offset}1},k_{\mathrm{offset}2}$)	$r_{\mathrm{top}}$	$r_{\mathrm{bottom}}$	*l*	*h*	$d_{0}$	$\lambda_{\mathrm{PS}1}$
([mm], [mm])	[mm]	[mm]	[mm]	[mm]	[mm]	[-]
$\left(0,0\right)$						
$\left(2.04,4\right)$	3	18	24	16	1	3
$\left(4.28,8\right)$						

**Table 3:** Parameter ranges used in the design.

Parameter		Range	Increment
$r_{top}$	[mm]	$1\leq r_{top}\leq 3$	0.5
$r_{bottom}$	[mm]	$5\leq r_{bottom}\leq 10$	1
*h*	[mm]	7≤h≤17	1
$k_{offset2}$	[mm]	$0\leq k_{offset2}\leq 8$	1
*l*	[mm]	10≤l≤15	1
$\lambda_{PS1}$	[-]	$2\leq \lambda_{PS1}\leq 4$	0.5

**Table 4:** Designed modules for the fish robot.

	← Head					Tail →		
Location			0.38 BL	0.41 BL	0.45 BL	0.48 BL	0.52 BL	0.55 BL
Module Number			➅	➄	➃	➂	➁	➀
Parameters								
	$r_{top}$	[mm]	2.5	2.5	2.5	2.5	2.5	2.5
	$r_{bottom}$	[mm]	9	9	9	8	8	7
	*h*	[mm]	9	9	9	9	9	9
	$k_{offset2}$	[mm]	2	4	4	4	3	2
	*l*	[mm]	14	14	14	14	13	12
	$\lambda_{PS1}$	[-]	4	4	3.5	3	3	2.5
Performance								
	$\tau_{0}$	[Nmm]	9.0	8.2	6.3	5.5	5.3	4.3
	θ	[deg]	6.6	7.2	7.4	5.5	5.8	4.3

## Data Availability

The original contributions presented in the study are included in the article, and further inquiries can be directed to the corresponding author.
